# Supplementary material for: OCTA measurements in Behcet’s disease across different stages of the disease activity: A systematic review and meta-analysis
Source: PLoS One. 2025 Jul 2;20(7):e0323192. doi: 10.1371/journal.pone.0323192 (PMC12221079; doi:10.1371/journal.pone.0323192)
Supplement: S1 File — (PDF) [file pone.0323192.s001.pdf]

OCTA measurements in Behcet's disease across different stages of the disease activity: A systematic review and meta-analysis

Authors: Mehrdad Mozafar[1,2 ¶ *], Mobina Amanollahi[1,3 ¶], Reza Samiee[1,3], Melika Jameie[4, 5], Ali Mousavi[6], Zahra Ghanbari[1], Helia Nafar[1], Negar Mozafar[7], Fatemeh Amiri[1], Mehdi Azizmohammad Looha[8], Elias Khalili Pour[3 *], Nazanin Ebrahimiadib[9]

1. School of Medicine, Tehran University of Medical Sciences, Tehran, Iran
2. Division of Vascular and Endovascular Surgery, Department of Surgery, Shohada-Tajrish Medical Center, Shahid Beheshti University of Medical Sciences, Tehran, Iran
3. Eye Research Center, Farabi Eye Hospital, Tehran University of Medical Sciences, Tehran, Iran
4. Neuroscience Research Center, Iran University of Medical Sciences, Tehran, Iran
5. Iranian Center of Neurological Research, Neuroscience Institute, Tehran University of Medical Sciences, Tehran, Iran
6. School of Medicine, Tabriz University of Medical Sciences, Tabriz, Iran
7. School of Medicine, Shahid Beheshti University of Medical Sciences, Tehran, Iran
8. Basic and Molecular Epidemiology of Gastrointestinal Disorders Research Center, Research Institute for Gastroenterology and Liver Diseases, Shahid Beheshti University of Medical Sciences, Tehran, Iran
9. Department of Ophthalmology, University of Florida, College of Medicine, Gainesville, FL, USA

¶ Mehrdad Mozafar and Mobina Amanollahi carry a co-first author status.

* Corresponding authors: Elias Khalili Pour and Mehrdad Mozafar share the correspondence.

Email: ekhalilipour@gmail.com, (EK)

Email: mehrdad.mozafar98@gmail.com, (MM)

Elias Khalili Pour, MD: Retina ward, Farabi Eye Hospital, South Kargar Street, Qazvin Square, Tehran, Iran.

Email: ekhalilipour@gmail.com, Tel: 0098-55410009, Fax: 0098-55421020

Mehrdad Mozafar, MD: Pour Sina Street, Tehran University of Medical Sciences, Tehran, Iran

Email: mehrdad.mozafar98@gmail.com, Tel:0098-9127020694

Supplementary Materials

Table of Contents		Page
**Table S1**	PubMed search strategy	**2**
**Table S2**	Web of Science search strategy	**3**
**Table S3**	Scopus search strategy	**4**
**Supplemental Results**		
**Active BU vs. HC**	Qualitative synthesis for studies not quantitatively pooled (Active BU vs. HC)	**5 - 6**
**Inactive BU vs. HC**	Qualitative synthesis for studies not quantitatively pooled (Inactive BU vs. HC)	**7 - 8**
**Active BU vs. inactive BU**	Qualitative synthesis for studies not quantitatively pooled (Active BU vs. inactive BU)	**9**
**Table S4**	Risk of bias assessment of the cross-sectional studies	**10 - 11**
**Table S5**	The PRISMA2020 statement: An updated guideline for reporting systematic reviews	**12 - 14**
**Table S6**	The PRISMA 2020 for Abstracts Checklist	**15**
**References**	-	**16**

Abbreviations: BU, Behcet Uveitis; HC, Healthy controls; BD, Behcet disease.

Table S1. PubMed Search Strategy, 10/9/2024: 45 results

Step	Syntax	Number of Results
**#1 (Behcet keywords)**	(“angiographic optical coherence tomography” [Title/Abstract] OR "optical coherence tomography angiography"[Title/Abstract] OR "OCTA"[Title/Abstract] OR "OCT-A"[Title/Abstract] OR "oct angiography"[Title/Abstract] OR "optical coherence tomographic angiography"[Title/Abstract])	13,856
**#2 (OCTA keywords)**	(Behcet [Title/Abstract] OR “Silk Road” [Title/Abstract] OR "Behcet Syndrome"[Mesh])	13,367
**#1 AND #2**	(“angiographic optical coherence tomography” [Title/Abstract] OR "optical coherence tomography angiography"[Title/Abstract] OR "OCTA"[Title/Abstract] OR "OCT-A"[Title/Abstract] OR "oct angiography"[Title/Abstract] OR "optical coherence tomographic angiography"[Title/Abstract]) AND (Behcet [Title/Abstract] OR "Behcet Syndrome"[Mesh])	45

Abbreviations: OCTA: Optical coherence tomography angiography

Table S2. Web of Science Search Strategy, 10/9/2024: 54 results

Number	Syntax	Number of the results
**#1 (Behcet keywords)**	(TS= (Behcet) OR TS= (“Silk Road”))	15,826
**#2 (OCTA keywords)**	(TS=(“angiographic optical coherence tomography”) OR TS=("optical coherence tomography angiography") OR TS=("OCTA") OR TS=("OCT-A") OR TS=("oct angiography") OR TS=("optical coherence tomographic angiography"))	15,761
**#1 AND #2**	(TS= (“angiographic optical coherence tomography”) OR TS=("optical coherence tomography angiography") OR TS=("OCTA") OR TS=("OCT-A") OR TS=("oct angiography") OR TS=("optical coherence tomographic angiography")) AND (TS= (Behcet) OR TS= (“Silk Road”))	54

Abbreviations: OCTA: Optical coherence tomography angiography

Table S3. Scopus Search Strategy, 10/9/2024: 81 results

Number	Syntax	Number of the results
**#1 (Behcet keywords)**	(TITLE-ABS-KEY(Behcet) OR TITLE-ABS-KEY(“Silk Road”) OR INDEXTERMS ("Behcet Syndrome"))	24,253
**#2 (OCTA keywords)**	(TITLE-ABS-KEY (“angiographic optical coherence tomography”) OR TITLE-ABS-KEY ("optical coherence tomography angiography") OR TITLE-ABS-KEY ("OCTA") OR TITLE-ABS-KEY ("OCT-A") OR TITLE-ABS-KEY ("oct angiography") OR TITLE-ABS-KEY ("optical coherence tomographic angiography"))	23,318
**#1 AND #2**	(TITLE-ABS-KEY (“angiographic optical coherence tomography”) OR TITLE-ABS-KEY ("optical coherence tomography angiography") OR TITLE-ABS-KEY ("OCTA") OR TITLE-ABS-KEY ("OCT-A") OR TITLE-ABS-KEY ("oct angiography") OR TITLE-ABS-KEY ("optical coherence tomographic angiography")) AND (TITLE-ABS-KEY(Behcet) OR TITLE-ABS-KEY(“Silk Road”) OR INDEXTERMS ("Behcet Syndrome"))	81

Abbreviations: OCT-A: Optical coherence tomography angiography

>>> Total records: 180

>>> Remaining after removing duplicates: 98

Supplemental Results: Active BU vs. HC

Whole (3*3) SRCP VD (active BU vs. HC)

Using the PLEX Elite device, Accorinti et al. (8 active BU and 15 HC eyes) found a significant decrease (P value = 0.001) in the whole (3*3) SRCP in the active BU group (0.432 ± 0.009) compared to the HCs (0.486 ± 0.004) [1]. However, Khairallah et al. (44 active BU and 22 HC eyes) applying the Topcon device, did not find a significant difference (P value = 0.167) between the active BU (11.7 ± 2.8) and HC (13.2 ± 1.6) groups [2].

Whole (6*6) SRCP VD (active BU vs. HC)

Accorinti et al. (8 active BU and 15 HC eyes) used PLEX Elite device, and found a significant decrease (P value = 0.007) in the whole (6*6) SRCP in the active BU group (0.460 ± 0.003) compared to the HCs (0.475 ± 0.002) [1]. Similarly, using RTVue XR Avanti device, Shen et al. found significantly lower parameter (P value = 0.003) in active BU (45.21 ± 3.68) compared to the control (48.97 ± 3.88) [3].

Parafoveal SRCP VD (active BU vs. HC)

Smid et al. (21 active BU and 22 HC eyes) using Heidelberg device, revealed a statistically significant decrease in the parafoveal SRCP VD (30 ± 9) in the active BU group compared to the HCs (38.9 ± 1.6) [4].

Whole (3*3) DRCP VD (active BU vs. HC)

Accorinti et al. (8 active BU and 15 HC eyes) used PLEX Elite device, and found a significant decrease (P value < 0.001) in the whole (3*3) DRCP in the active BU group (0.436 ± 0.009) compared to the HCs (0.496 ± 0.005) [1]. Moreover, Khairallah et al. (44 active BU and 22 HC eyes) applying the Topcon device, detected a significant decrease (P value = 0.004) in the active BU (13.7 ± 4) compared to the HC (17.2 ± 1.4) group [2].

Whole (6*6) DRCP VD (active BU vs. HC)

Accorinti et al. (8 active BU and 15 HC eyes) used the PLEX Elite device, and found a significant decrease (P value = 0.003) in the whole (6*6) DRCP in the active BU group (0.458 ± 0.005) compared to the HCs (0.478 ± 0.003) [1]. In the same way, Shen et al. found significantly lower whole SRCP (6*6) (P value = 0.04) in active BU (45.58 ± 4.61) compared to the control (48.25 ± 6.60).

Parafoveal DRCP VD (active BU vs. HC)

Smid et al. (21 active BU and 22 HC eyes) using the Heidelberg device, revealed a statistically significant decrease in the parafoveal DRCP VD (25 ± 7) in the active BU group compared to the HCs (33.5 ± 1.9) [4].

Choriocapillaris flow area (active BU vs. HC)

Yan et al. (24 active BU and 24 HC eyes) compared choriocapillaris (1 mm) between active BU and HC subgroups using the RTVue XR Avanti device [5]. Accordingly, the choriocapillaris flow area was significantly lower in BUactive eyes (1.91 ± 0.24) than HCs (2.37 ± 0.13) (P = 0.009). Using the same device, Shen et al. assessed choriocapillaris in 3 mm diameter in 25 active BU cases and 416 HC [3]. They found significantly lower VD in the metric among patients as compared to the controls (P value= 0.004).

RPC VD (active BU vs. HC)

Shen et al. applied RTVue XR Avanti device to measure RPC VD, and found significantly reduced parameter (P value = 0.01) in active BU (53.97 ± 4.79) compared to the control (56.35 ± 2.43) [3].

FAZ (active BU vs. HC)

Using RTVue XR Avanti device, Shen et al. found no significant difference (P value = 0.93) in active BU (0.32 ± 0.07) compared to the control (0.33 ± 0.13) [3].

FAZ superficial (active BU vs. HC)

Khairallah et al. (38 active BU (for FAZ parameter) and 22 HC eyes) compared FAZ (superficial) between active BU and HC subgroups using Topcon device [2]. Accordingly, there was no statistically significant (P value = 0.23) difference between the active BU group (0.4 ± 0.15) and HCs (0.34 ± 0.88).

FAZ deep (active BU vs. HC)

Khairallah et al. (44 active BU and 22 HC eyes) compared FAZ (deep) between active BU and HC subgroups using the Topcon device [2]. There was no statistically significant (P value = 0.053) difference in the FAZ (deep) size between the active BU group (0.72 ± 0.25) and HCs (0.53 ± 0.16).

Supplemental Results: Inactive BU vs. HC

Whole (3*3) SRCP VD (inactive BU vs. HC)

Accorinti et al. (15 inactive BU and 15 HC eyes) utilized the PLEX Elite device, and found no significant difference (P value = 1.000) in the whole (3*3) SRCP VD between the inactive BU group (0.476 ± 0.007) and the HCs (0.486 ± 0.004) [1]. On the other hand, Ferreira et al. (23 inactive BU and 26 HC eyes) using the Heidelberg device, found a significant lower whole (3*3) SRCP in the inactive BU group (0.231 ± 0.028) compared to HCs (0.253 ± 0.019) [6].

Whole (6*6) SRCP VD (inactive BU vs. HC)

Accorinti et al. (15 inactive BU and 15 HC eyes) utilized the PLEX Elite device, and found no significant difference (P value = 0.113) in the whole (6*6) SRCP VD between the inactive BU group (0.467 ± 0.003) and the HCs (0.475 ± 0.002) [1].

Foveal SRCP VD (inactive BU vs. HC)

Dai et al. (52 inactive BU and 50 HC eyes) using the Svision machine, reported significantly reduced (P value = 0.006) the foveal SRCP VD in inactive BU than HC eyes [7].

Parafoveal SRCP VD (inactive BU vs. HC)

Eser Ozturk et al. (42 inactive BU and 38 HC eyes) using the Topcon machine, reported no significant (P value = 0.520) difference in the parafoveal SRCP VD between inactive BU (20.89 ± 5.49) and HC (21.58 ± 3.82) eyes [8].

DRCP whole (3*3) VD (inactive BU vs. HC)

Accorinti et al. (15 inactive BU and 15 HC eyes) utilized the PLEX Elite device, and found a significant decrease (P value = 0.030) in the whole (3*3) DRCP VD in the inactive BU group (0.464 ± 0.009) compared to the HCs (0.496 ± 0.005) [1]. Furthermore, Ferreira et al. (23 inactive BU and 26 HC eyes) using the Heidelberg device, found a significant lower whole (3*3) DRCP in the inactive BU group (0.263 ± 0.03) compared to HCs (0.315 ± 0.03) [6].

Whole (6*6) DRCP VD (inactive BU vs. HC)

Accorinti et al. (15 inactive BU and 15 HC eyes) utilized the PLEX Elite device, and found no significant difference (P value = 0.343) in the whole (6*6) DRCP VD between the inactive BU group (0.471 ± 0.004) and the HCs (0.478 ± 0.003) [1].

Foveal DRCP VD **(inactive BU vs. HC)**

Dai et al. (52 inactive BU and 50 HC eyes) used the Svision machine, and reported significantly reduced (P value = 0.01) the foveal DRCP VD in inactive BU than HC eyes [7].

Parafoveal DRCP VD **(inactive BU vs. HC)**

Eser Ozturk et al. (42 inactive BU and 38 HC eyes) using the Topcon machine, reported no significant (P value = 0.532) difference in the parafoveal SRCP VD between inactive BU (21.17 ± 5.46) and HC (21.71 ± 3.88) eyes [8].

Choriocapillaris flow area **(inactive BU vs. HC)**

Aksoy et al. (35 active BU and 30 HC eyes) compared choriocapillaris (3 mm) between inactive BU and HC subgroups using the RTVue XR Avanti device [9]. Accordingly, the choriocapillaris flow area was not significantly different between inactive BU eyes (16.78 ± 1.24) and HCs (17.39 ± 1.41) (P = 0.065).

FAZ superficial **(inactive BU vs. HC)**

Guo et al. (57 inactive BU and 60 HC eyes), using BM-400K BMizar device, showed that FAZ superficial was significantly larger in the inactive BU eyes compared with HCs (P value = 0.005) [10].

FAZ deep **(inactive BU vs. HC)**

Guo et al. (57 inactive BU and 60 HC eyes), using BM-400K BMizar device, showed that FAZ deep was significantly larger in the inactive BU eyes compared with HCs (p value = 0.05) [10].

Supplemental Results: Active BU vs. inactive BU

Whole (3*3) SRCP (active BU vs. inactive BU)

Accorinti et al. (8 active BU and 15 inactive BU eyes) utilized the PLEX Elite device and found a significant decrease (P value = 0.004) in the whole (3*3) SRCP VD in the active BU group (0.432 ± 0.009) than the inactive BU group (0.476 ± 0.007) [1].

Whole (6*6) SRCP (active BU vs. inactive BU)

Accorinti et al. (8 active BU and 15 inactive BU eyes) utilized the PLEX Elite device and found no significant difference (P value = 0.425) in the whole (6*6) SRCP VD between the active BU (0.460 ± 0.003) and the inactive BU groups (0.467 ± 0.003) [1].

Whole (3*3) DRCP (active BU vs. inactive BU)

Accorinti et al. (8 active BU and 15 inactive BU eyes) utilized the PLEX Elite device and found no significant difference (P value = 0.212) in the whole (3*3) DRCP VD between the active BU (0.436 ± 0.009) and the inactive BU groups (0.464 ± 0.009) [1].

Whole (6*6) DRCP (active BU vs. inactive BU)

Accorinti et al. (8 active BU and 15 inactive BU eyes) utilized the PLEX Elite device and found no significant difference (P value = 0.090) in the whole (6*6) DRCP VD between the active BU (0.458 ± 0.005) and the inactive BU groups (0.471 ± 0.004) [1].

Table S4. Risk of bias assessment of the cross-sectional studies using the Newcastle-Ottawa scale (NOS) modified for cross-sectional studies.

Study First author (year)	Selection					Comparability			Exposure				Total score
	Case definition adequacy	Representative of cases	Selection of controls	Definition of controls	Subtotal	Age	Sex	Subtotal	Ascertainment of exposure	Same ascertainment	Non-response rate	Subtotal	
**Accorinti et al. (2019)**	*	*	-	*	3	*	*	2	*	*	*	3	8
**Aksoy et al. (2020)**	*	*	-	*	3	*	*	2	*	*	*	3	8
**Balicoglu Yilmaz et al. (2020)**	*	*	-	-	2	-	-	0	*	*	*	3	5
**Cheng et al. (2018)**	*	*	-	*	3	*	*	2	*	*	*	3	8
**Comez et al. (2019)**	*	*	-	*	3	-	-	0	*	*	*	3	6
**Dai et al. (2024)**	*	*	*	*	4	*	*	2	*	*	*	3	9
**Degirmenci et al. (2018)**	*	-	-	-	1	*	*	2	*	*	*	3	6
**Emre et al. (2019)**	*	*	-	-	2	-	-	0	*	*	*	3	5
**Eser-Ozturk et al. (2021)**	*	*	*	*	4	*	-	1	*	*	*	3	8
**Ferreira et al. (2023)**	*	*	*	*	4	*	*	2	*	*	*	3	9
**Goker et al. (2019)**	*	*	*	*	4	*	*	2	*	*	*	3	9
**Guo et al. (2023)**	*	*	*	*	4	*	*	2	*	*	*	3	9
**Kianersi et al. (2024)**	*	-	*	*	3	*	*	2	*	*	*	3	8

**Khairallah et al. (2017)**	*	*	-	-	2	*	-	1	*	*	*	3	6
**Koca et al. (2019)**	*	*	*	*	4	-	-	0	*	*	*	3	7
**Karalezli et al. (2021)**	*	*	-	*	3	*	*	2	*	*	*	3	8
**Küçük et al. (2022)**	*	*	*	*	4	*	*	2	*	*	*	3	9
**Karaca et al. (2023)**	-	*	-	*	2	-	-	0	*	*	*	3	5
**Nassar et al. (2022)**	*	*	-	-	2	*	*	2	*	*	*	3	7
**Pei et al. (2019)**	*	*	-	-	2	-	-	0	*	*	*	3	5
**Raafat et al. (2019)**	*	*	-	-	2	*	-	1	*	*	*	3	6
**Shen et al. (2024)**	*	-	*	*	3	*	-	1	*	*	*	3	7
**Smid et al. (2021)**	*	*	*	*	4	*	*	2	*	*	*	3	9
**Simsek et al. (2022)**	*	*	*	*	4	*	*	2	*	*	*	3	9
**Türkcü et al. (2020)**	-	*	-	*	2	-	-	0	*	*	*	3	5
**Yan et al. (2021)**	*	*	-	-	2	*	*	2	*	*	*	3	7
**Yilmaz et al. (2021)**	*	*	*	*	4	-	-	0	*	*	*	3	7
**Yılmaz Tuğan et al. (2022)**	*	*	*	*	4	-	-	0	*	*	*	3	7

Abbreviations: NOS: Newcastle-Ottawa scale

Table S5. The PRISMA2020 statement: An updated guideline for reporting systematic reviews.

Section and Topic	Item #	Checklist item	Location where item is reported
**TITLE**			
Title	1	Identify the report as a systematic review.	Page 1
**ABSTRACT**			
Abstract	2	See the PRISMA 2020 for Abstracts checklist.	Table S6
**INTRODUCTION**			
Rationale	3	Describe the rationale for the review in the context of existing knowledge.	Page 3
Objectives	4	Provide an explicit statement of the objective(s) or question(s) the review addresses.	Page 3
**METHODS**			
Eligibility criteria	5	Specify the inclusion and exclusion criteria for the review and how studies were grouped for the syntheses.	Page 4
Information sources	6	Specify all databases, registers, websites, organisations, reference lists and other sources searched or consulted to identify studies. Specify the date when each source was last searched or consulted.	Page 4
Search strategy	7	Present the full search strategies for all databases, registers and websites, including any filters and limits used.	Page 4, AND Tables S1-S3
Selection process	8	Specify the methods used to decide whether a study met the inclusion criteria of the review, including how many reviewers screened each record and each report retrieved, whether they worked independently, and if applicable, details of automation tools used in the process.	Page 4, Prisma figure (Figure 1)
Data collection process	9	Specify the methods used to collect data from reports, including how many reviewers collected data from each report, whether they worked independently, any processes for obtaining or confirming data from study investigators, and if applicable, details of automation tools used in the process.	Pages 5
Data items	10a	List and define all outcomes for which data were sought. Specify whether all results that were compatible with each outcome domain in each study were sought (e.g. for all measures, time points, analyses), and if not, the methods used to decide which results to collect.	Page 5-7
	10b	List and define all other variables for which data were sought (e.g. participant and intervention characteristics, funding sources). Describe any assumptions made about any missing or unclear information.	Page 5-7
Study risk of bias assessment	11	Specify the methods used to assess risk of bias in the included studies, including details of the tool(s) used, how many reviewers assessed each study and whether they worked independently, and if applicable, details of automation tools used in the process.	Page 8
Effect measures	12	Specify for each outcome the effect measure(s) (e.g. risk ratio, mean difference) used in the synthesis or presentation of results.	Page 8
Synthesis methods	13a	Describe the processes used to decide which studies were eligible for each synthesis (e.g. tabulating the study intervention characteristics and comparing against the planned groups for each synthesis (item #5)).	Page 8
	13b	Describe any methods required to prepare the data for presentation or synthesis, such as handling of missing summary statistics, or data conversions.	Page 8

Section and Topic	Item #	Checklist item	Location where item is reported
	13c	Describe any methods used to tabulate or visually display results of individual studies and syntheses.	Page 8
	13d	Describe any methods used to synthesize results and provide a rationale for the choice(s). If meta-analysis was performed, describe the model(s), method(s) to identify the presence and extent of statistical heterogeneity, and software package(s) used.	Page 8
	13e	Describe any methods used to explore possible causes of heterogeneity among study results (e.g. subgroup analysis, meta-regression).	Page 8
	13f	Describe any sensitivity analyses conducted to assess robustness of the synthesized results.	-
Reporting bias assessment	14	Describe any methods used to assess risk of bias due to missing results in a synthesis (arising from reporting biases).	-
Certainty assessment	15	Describe any methods used to assess certainty (or confidence) in the body of evidence for an outcome.	-
**RESULTS**			
Study selection	16a	Describe the results of the search and selection process, from the number of records identified in the search to the number of studies included in the review, ideally using a flow diagram.	Page 9-30
	16b	Cite studies that might appear to meet the inclusion criteria, but which were excluded, and explain why they were excluded.	-
Study characteristics	17	Cite each included study and present its characteristics.	Page 9-30
Risk of bias in studies	18	Present assessments of risk of bias for each included study.	Page 42
Results of individual studies	19	For all outcomes, present, for each study: (a) summary statistics for each group (where appropriate) and (b) an effect estimate and its precision (e.g. confidence/credible interval), ideally using structured tables or plots.	Page 31-41<br>Note: Since the number of outcomes in the study is high, they are described in several subheadings from pages 31 to 41. Also, those studies in which meta-analyses could not be conducted were incorporated in the Supplementary Materials
Results of syntheses	20a	For each synthesis, briefly summarise the characteristics and risk of bias among contributing studies.	Page 31-41<br>Note: Since the number of outcomes in the study is high, they are described in several subheadings from pages 31 to 41. Also, those studies in which meta-analyses could not be conducted were incorporated in the Supplementary Materials

Section and Topic	Item #	Checklist item	Location where item is reported
	20b	Present results of all statistical syntheses conducted. If meta-analysis was done, present for each the summary estimate and its precision (e.g. confidence/credible interval) and measures of statistical heterogeneity. If comparing groups, describe the direction of the effect.	Page 31-41<br>Note: Since the number of outcomes in the study is high, they are described in several subheadings from pages 31 to 41. Also, those studies in which meta-analyses could not be conducted were incorporated in the Supplementary Materials
	20c	Present results of all investigations of possible causes of heterogeneity among study results.	Page 31-41
	20d	Present results of all sensitivity analyses conducted to assess the robustness of the synthesized results.	-
Reporting biases	21	Present assessments of risk of bias due to missing results (arising from reporting biases) for each synthesis assessed.	-
Certainty of evidence	22	Present assessments of certainty (or confidence) in the body of evidence for each outcome assessed.	-
**DISCUSSION**			
Discussion	23a	Provide a general interpretation of the results in the context of other evidence.	Page 42
	23b	Discuss any limitations of the evidence included in the review.	Page 42
	23c	Discuss any limitations of the review processes used.	Page 43
	23d	Discuss implications of the results for practice, policy, and future research.	Page 43
**OTHER INFORMATION**			
Registration and protocol	24a	Provide registration information for the review, including register name and registration number, or state that the review was not registered.	Page 4
	24b	Indicate where the review protocol can be accessed, or state that a protocol was not prepared.	Page 4
	24c	Describe and explain any amendments to information provided at registration or in the protocol.	-
Support	25	Describe sources of financial or non-financial support for the review, and the role of the funders or sponsors in the review.	Page 45
Competing interests	26	Declare any competing interests of review authors.	Page 45
Availability of data, code and other materials	27	Report which of the following are publicly available and where they can be found: template data collection forms; data extracted from included studies; data used for all analyses; analytic code; any other materials used in the review.	-

From: Page MJ, McKenzie JE, Bossuyt PM, Boutron I, Hoffmann TC, Mulrow CD, et al. The PRISMA 2020 statement: an updated guideline for reporting systematic reviews. BMJ 2021;372:n71. doi: 10.1136/bmj.n71

For more information, visit: http://www.prisma-statement.org/

Table S6. The PRISMA 2020 for Abstracts Checklist

Section and Topic	Item #	Checklist item	Reported (Yes/No)
**TITLE**			
Title	1	Identify the report as a systematic review.	Yes
**BACKGROUND**			
Objectives	2	Provide an explicit statement of the main objective(s) or question(s) the review addresses.	Yes
**METHODS**			
Eligibility criteria	3	Specify the inclusion and exclusion criteria for the review.	Yes
Information sources	4	Specify the information sources (e.g. databases, registers) used to identify studies and the date when each was last searched.	Yes
Risk of bias	5	Specify the methods used to assess risk of bias in the included studies.	Yes
Synthesis of results	6	Specify the methods used to present and synthesise results.	Yes
**RESULTS**			
Included studies	7	Give the total number of included studies and participants and summarise relevant characteristics of studies.	Yes
Synthesis of results	8	Present results for main outcomes, preferably indicating the number of included studies and participants for each. If meta-analysis was done, report the summary estimate and confidence/credible interval. If comparing groups, indicate the direction of the effect (i.e. which group is favoured).	Yes
**DISCUSSION**			
Limitations of evidence	9	Provide a brief summary of the limitations of the evidence included in the review (e.g. study risk of bias, inconsistency and imprecision).	Yes
Interpretation	10	Provide a general interpretation of the results and important implications.	Yes
**OTHER**			
Funding	11	Specify the primary source of funding for the review.	No
Registration	12	Provide the register name and registration number.	No

From: Page MJ, McKenzie JE, Bossuyt PM, Boutron I, Hoffmann TC, Mulrow CD, et al. The PRISMA 2020 statement: an updated guideline for reporting systematic reviews. BMJ 2021;372:n71. doi: 10.1136/bmj.n71

References

1. Accorinti, M., et al., *Optical Coherence Tomography Angiography Findings in Active and Inactive Ocular Behçet Disease.* Ocul Immunol Inflamm, 2020. **28**(4): p. 589-600.
2. Khairallah, M., et al., *OPTICAL COHERENCE TOMOGRAPHY ANGIOGRAPHY IN PATIENTS WITH BEHÇET UVEITIS.* Retina, 2017. **37**(9): p. 1678-1691.
3. Junhui, S., Q. Guangming, and F. Lei, *Lipid profile alterations in non-infectious uveitis: correlation with quantitative optical coherence tomography angiography parameters.* BMJ Open Ophthalmology, 2024. **9**(1): p. e001641.
4. Smid, L.M., et al., *Parafoveal Microvascular Alterations in Ocular and Non-Ocular Behçet's Disease Evaluated With Optical Coherence Tomography Angiography.* Invest Ophthalmol Vis Sci, 2021. **62**(3): p. 8.
5. Yan, C., et al., *Vascular Abnormalities in Peripapillary and Macular Regions of Behcet's Uveitis Patients Evaluated by Optical Coherence Tomography Angiography.* Front Med (Lausanne), 2021. **8**: p. 727151.
6. Ferreira, B.F.A., et al., *OPTICAL COHERENCE TOMOGRAPHY ANGIOGRAPHY BIOMARKERS AND MICROPERIMETRY FEATURES IN BEHÇET'S UVEITIS.* Retina, 2023. **43**(10): p. 1680-1690.
7. Dai, L., et al., *Sensitive Optical Coherence Tomography Angiography Parameters Detecting Retinal Vascular Changes in Behcet's Uveitis.* Photodiagnosis and Photodynamic Therapy, 2024: p. 104353.
8. Eser-Ozturk, H., et al., *Quantitative measurements with optical coherence tomography angiography in Behçet uveitis.* Eur J Ophthalmol, 2021. **31**(3): p. 1047-1055.
9. Aksoy, F.E., et al., *Retinal microvasculature in the remission period of Behcet's uveitis.* Photodiagnosis Photodyn Ther, 2020. **29**: p. 101646.
10. Guo, S., et al., *ANALYSIS OF VASCULAR CHANGES OF FUNDUS IN BEHCET UVEITIS BY WIDEFIELD SWEPT SOURCE OPTICAL COHERENCE TOMOGRAPHY ANGIOGRAPHY AND FUNDUS FLUORESCEIN ANGIOGRAPHY.* Retina, 2023. **43**(5): p. 841-850.
