# Supplementary material for: OCTA measurements in Behcet’s disease across different stages of the disease activity: A systematic review and meta-analysis
Source: PLoS One. 2025 Jul 2;20(7):e0323192. doi: 10.1371/journal.pone.0323192 (PMC12221079; doi:10.1371/journal.pone.0323192)
Supplement: Attachment — Submitted filename: Response to Reviewers.docx [file pone.0323192.s006.docx]

**PONE-D-24-46800**

**OCTA measurements in Behcet’s disease across different stages of the disease activity: A systematic review and meta-analysis**

**PLOS ONE**

**Reply**: Dear Dr. Elnahry,

Thank you very much for providing us with the opportunity to strengthen our manuscript. Having carefully considered the comments and suggestions, we have made all the relevant changes to our manuscript as outlined below in an itemized, point-by-point manner. We sincerely hope that these changes meet the approval criteria of the esteemed reviewers and the editorial board.

Best Regards,

Elias Khalili Pour M.D,

Retina ward, Farabi Eye Hospital,

South Kargar Street, Qazvin Square, Tehran, Iran.

ekhalilipour@gmail.com

**==========================================================================Response to the comments of the Journal Requirement:**

**Dear Authors,
You have done a large amount of work, which I appreciate. Some issues need to be improved.**

- **Comment #1: Please include a caption for figure 9.**
- **Reply**: Thank you so much for bringing this to our attention. It is added now.

**==========================================================================Response to the comments of the Esteemed Associate Editor:**

- **Comment #1: The authors report an extensive systematic review and meta analysis of studies that assessed different groups with BD using OCTA. The analysis is extensive and comprehensive and the authors should be congratulated on this. The main issue is the multiple comparisons that the authors underwent due to the heterogeneity of the studies. The authors should therefore perform p-value corrections for their analyses such as Bonferroni correction. When conducting a statistical analysis that involves multiple comparisons, such as comparing multiple subgroups, the Bonferroni correction can be applied to reduce the risk of false positives. I recommend the authors do this correction to all their analyses including comparisons and meta analyses results. The authors should revise the discussion section accordingly as needed.**
- **Response:** We sincerely appreciate your thoughtful feedback and your recognition of the comprehensiveness of our systematic review and meta-analysis. Your suggestion regarding multiple comparisons and the application of **p-value corrections** is highly valuable, and we have carefully addressed this concern in our revised analysis.
- **Implementation of Multiple Comparison Correction**
- To account for the increased risk of **false positives** due to multiple comparisons, we applied the **False Discovery Rate (FDR) correction using the Benjamini-Hochberg (BH) method** rather than the Bonferroni correction. FDR correction is more appropriate for meta-analyses because it controls the expected proportion of false discoveries rather than strictly limiting Type I errors, which can be overly conservative in large-scale analyses.
- The Benjamini-Hochberg procedure follows these steps:
- **Rank all p-values** from smallest to largest across the multiple comparisons being made.
- **Calculate the critical value** for each p-value using the formula:
- $\alpha_{adjusted,i}= \frac{i}{m}\times\alpha$
- where:
- i is the rank of the p-value,
- m is the total number of comparisons, and
- $\alpha$ is the desired false discovery rate (typically set at 0.05).
- **Compare each p-value to its corresponding critical value.** The largest p-value that is still below its critical value is considered significant, and all smaller p-values are also deemed significant.
- **Adjust the p-values accordingly** to ensure that they are monotonically increasing, preserving the correct ordering.
- Additionally, we implemented the FDR correction using R, applying the p.adjust() function with the Benjamini-Hochberg method, as follows:
- ----
- # Example list of uncorrected p-values from our meta-analysis
- p_values <- c(0.045, 0.009, 0.021, 0.031, 0.002, 0.056, 0.015)
- # Apply Benjamini-Hochberg (FDR) correction
- adjusted_p_values <- p.adjust(p_values, method = "BH")
- # Print results
- data.frame(Uncorrected_P = p_values, Adjusted_P = adjusted_p_values)
- ----
- **Application of Correction to Results**
- We have systematically re-evaluated all statistical comparisons and **updated the p-values** in our results accordingly. Specifically:
- **All p-values from subgroup comparisons and meta-analyses have been adjusted** using the Benjamini-Hochberg correction.
- We have carefully **updated the results and p-values**, ensuring that both raw and corrected values are now included in the manuscript. Notably, none of the previously significant raw p-values lost their significance after correction. Therefore, the discussion and main text of the results remain unchanged in this regard.
- A statement has been added to the **method section**, mentioning our approach for correcting for multiple comparisons.
- **A new table (Table 4) has been added** to the manuscript, where we now report the meta-analyses results along with both the **uncorrected p-values and the corrected p-values** for each comparison across all outcomes. This provides clarity on how the adjustments impact statistical significance and ensures full transparency in our methodology.
- Thank you once again for your constructive feedback, which has significantly improved our manuscript.

===========================================================================**Response to the comments of the Reviewer 1:**

- **Comment #2: Very interesting study with sound methodology and statistics. Nice effort. My only comment would be that the conclusion could be more precise and reflecting the results. For example, the suggestion of screening eyes with non ocular Behcet’s lacks the correlation to management as screening will only be relevant if a particular intervention would be undertaken.**
- **Reply**: Thank you for your thoughtful feedback and for recognizing the strengths of our study. We appreciate your suggestion regarding the precision of the conclusion, particularly in relation to the recommendation for screening eyes in patients with non-ocular Behçet’s disease (BD).
- We acknowledge that screening is most impactful when it directly informs management decisions. Our intention was to highlight the presence of retinal microvascular changes in non-ocular BD, as observed in OCTA studies, which may indicate subclinical vascular involvement. However, we agree that the clinical implications of such findings require further clarification. To address this, we have refined our conclusion to ensure it accurately reflects the results while acknowledging the need for additional research to determine the potential utility of retinal screening in clinical decision-making.
- **==========================================================================**
- **Response to the comments of the Reviewer 2:**
- **Comment #1: Line 74 and line 128 any disagreements were solved by SHOULD be any disagreement was solved by.**
- **Reply**: Thank you very much for your precious comment. They are corrected grammatically.
- **Comment #2: Page 16 at the end senile SHOULD age-related macular degeneration**
- **Reply**: Thank you very much for your feedback. It is revised.
- **Comment #3: Page 17 top line glaucoma suspicion SHOULD be glaucoma suspect.**

**Reply**: Thank you very much. We have changed the wording to “suspect”.

- **Comment #4: Page 17 bottom. Dense media obstacles SHOULD be Dense media opacity**. **Reply**: Thank you very much. We have changed the wording to “Dense media opacity”.

**=========================================================================**

- **Comment #5: CONCLUSION: “Given that our analysis found no significant reduction in vessel density in cases of active BD”**

**Reason: patients on prior medical therapy but with flare up; degree of activity varies from mild to severe with mild not affecting vessel density and severe causing temporary occlusion; view too bad to allow visualization of vessels in severe uveitis precluding good quality OCTA (selection bias). Retinal vascular occlusions were found in a third of ocular BD patients.**

**Ostrovsky M, Ramon D, Iriqat S, Shteiwi A, Sharon Y, Kramer M, Vishnevskia-Dai V, Sar S, Boulos Y, Tomkins-Netzer O, Lavee N, Ben-Arie-Weintrob Y, Pizem H, Hareuveni-Blum T, Schneck M, Gepstein R, Masarwa D, Nakhoul N, Bakshi E, Shulman S, Goldstein M, Anouk M, Rosenblatt A, Habot-Wilner Z. Retinal vascular occlusions in ocular Behçet disease - a comparative analysis. Acta Ophthalmol. 2023 Sep;101(6):619-626.**

**Population bias: Risk factors for retinal vascular occlusions included Jewish ethnicity (p < 0.05).**

**Ostrovsky M, Ramon D, Iriqat S, Shteiwi A, Sharon Y, Kramer M, Vishnevskia-Dai V, Sar S, Boulos Y, Tomkins-Netzer O, Lavee N, Ben-Arie-Weintrob Y, Pizem H, Hareuveni-Blum T, Schneck M, Gepstein R, Masarwa D, Nakhoul N, Bakshi E, Shulman S, Goldstein M, Anouk M, Rosenblatt A, Habot-Wilner Z. Retinal vascular occlusions in ocular Behçet disease - a comparative analysis. Acta Ophthalmol. 2023 Sep;101(6):619-626.**

**Yahia, Salim Ben, Rim Kahloun, Bechir Jelliti, and Moncef Khairallah. 2011. “Branch Retinal Artery Occlusion Associated with Behçet Disease.” Ocular Immunology and Inflammation 19 (4): 293–95.**

- **Reply:** We appreciate the reviewer’s insightful observations regarding the potential reasons for the lack of significant reduction in vessel density in cases of active BD. In the limitation section of the discussion, we acknowledged that prior medical therapy, varying degrees of disease activity, and selection bias due to poor visualization in severe uveitis may have influenced the findings. Additionally, the presence of retinal vascular occlusions in a subset of BD patients could contribute to the heterogeneity of vascular changes observed in OCTA studies.
- To address these points, we have also revised the conclusion to better reflect these considerations and acknowledge the need for future studies to account for these confounding factors.
